# Accuracy of CAD/CAM Digital Impressions with Different Intraoral Scanner Parameters

**DOI:** 10.3390/s20041157

**Published:** 2020-02-20

**Authors:** Asher Chiu, Yen-Wei Chen, Juri Hayashi, Alireza Sadr

**Affiliations:** Department of Restorative Dentistry, University of Washington School of Dentistry, Seattle, WA 98195, USA

**Keywords:** digital impression, CAD/CAM, accuracy, intraoral scanner, high resolution

## Abstract

The advancement of intraoral scanners has allowed for more efficient workflow in the dental clinical setting. However, limited data exist regarding the accuracy of the digital impressions produced with various scanner settings and scanning approaches. The purpose of this in vitro study was to compare the accuracy of digital impressions at the crown preparation margin using different scanning resolutions of a specific intraoral scanner system. An all-ceramic crown preparation of a mandibular first molar was constructed in a typodont, and a scan (n = 3) was created with an industrial-grade laboratory scanner (3Shape D2000) as the control. Digital impressions were obtained with an intraoral scanner (3Shape TRIOS 3) under three settings—high resolution (HR), standard resolution (SR), and combined resolution (SHR). Comparative 3D analysis of scans was performed with Geomagic Control X software to measure the discrepancy between intraoral scans and the control scan along the preparation finish line. The scan time and number of images captured per scan were recorded. Statistical analysis was performed by one-way ANOVA, two-way repeated measures ANOVA, Pearson’s correlation, and Dunnett’s T3 test (α = 0.05). Significant differences were observed for scan time and for number of images captured among scan resolution settings (α < 0.05). The scan time for the SR group was, on average, 34.2 s less than the SHR group and 46.5 s less than the HR group. For discrepancy on the finish line, no significant differences were observed among scanning resolutions (HR: 31.5 ± 5.5 μm, SHR: 33.2 ± 3.7 μm, SR: 33.6 ± 3.1 μm). Significant differences in discrepancy were observed among tooth surfaces, with the distal surface showing the highest discrepancies. In conclusion, the resolution of the intra-oral scanner is primarily defined by the system hardware and optimized for default scans. A software high-resolution mode that obtains more data over a longer time may not necessarily benefit the scan accuracy, while the tooth preparation and surface parameters do affect the accuracy.

## 1. Introduction

Computer-aided design and computer-aided manufacturing (CAD/CAM) technology has drastically changed the face of dentistry since it was introduced to the field in the 1980s [1]. In the early stages of the application of CAD/CAM to dentistry, desktop scanners were used in dental laboratories to digitize gypsum models before the milling and manufacturing of dental prosthetics [2]. Most recently, the advancement of chairside CAD/CAM systems has provided a more efficient digital workflow in the clinical setting [3]. In the last two decades, many commercially available intraoral scanners (IOS) have been developed [4], and both in vivo and in vitro studies have examined the accuracy and precision of various intraoral scanners compared to conventional impression materials and techniques [5]. The use of intraoral scanners as an alternative to conventional impression reduces patient discomfort, is more environmentally friendly, and is easier for clinicians to manipulate without the risk of damage or distortion [6]. Other advantages of intraoral scanning include real-time visualization and magnification, automatic color-scanning for esthetic shade selection, and better patient compliance. Along with improved reliability and reproducibility in the technology, these advantages have increased the acceptance and popularity of digital impression [7].

The success of any dental restoration in the long term depends on its marginal adaptation to the existing tooth structure. Complications such as caries may arise around the margins as a result of bacterial penetration into leaking open margins and biofilm accumulation on marginal discrepancies. Therefore, obtaining an accurate impression from the tooth is critical in the fabrication process of a dental restoration [8].

**Figure 1:**
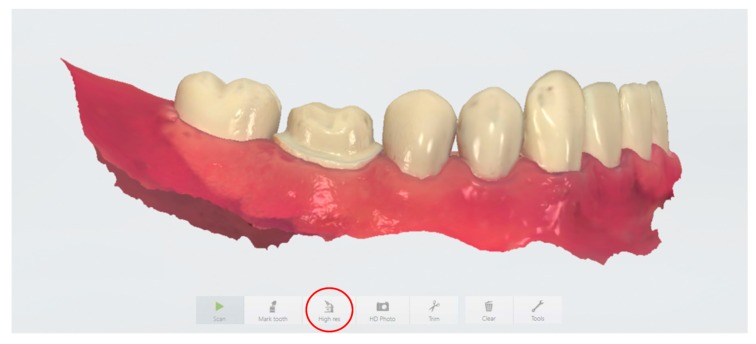
Depiction of the digital interface using the 3Shape TRIOS 3 scanner and software, highlighting the High Resolution (High res) feature.

Given that intraoral scanning is the first and therefore foundational step in chairside digital workflow, the accuracy of intraoral scanners must be evaluated critically. To this end, we examined the 3Shape TRIOS 3 intraoral scanner (3Shape, Copenhagen, Denmark) because it is one of the major intraoral scanning systems currently on the market and has gained widespread use in restorative dentistry. In addition, several recent studies have shown that the TRIOS 3 is one of the most accurate intraoral scanners, in comparison to other intraoral scanning systems [9,10,11]. However, different scan settings have been suggested by the manufacturer and a variety of scanning techniques have been applied depending on operator preferences. More specifically, the 3Shape TRIOS 3 user manual (ver. 2017) recommends the use of High Resolution (High res; also known as Zoom in some software versions) when scanning critical surfaces such as crown preparation margins in order to “capture areas that are difficult to scan with higher amounts of details” (Figure 1). Data on the accuracy of digital impressions made under different scanning resolution settings are insufficient, however. Theoretically, the High res function would allow for superior finish line accuracy to minimize marginal discrepancy between the preparation and the restoration. Clinically, however, using the High res function requires additional chair-time and can be disruptive to the provider’s workflow.

The purpose of this study was to investigate the difference in accuracy between digital impressions obtained using various scan resolution settings on the 3Shape TRIOS 3 scanner. These data allowed evaluation of the necessity of the TRIOS 3 scanner High res function as an essential step in taking digital impressions for single unit fixed dental prostheses. Furthermore, we were most interested in the accuracy of the scans at the cavosurface finish line on the prepared tooth, where restoration margin integrity is critical to the success and longevity of the restoration. The null hypothesis was that no significant difference in discrepancy was expected among different intraoral scanning resolutions.

## 2. Materials and Methods

### 2.1. Control Scan Preparation

A mandibular right first molar was prepared for an all-ceramic crown on a typodont (Columbia Dentoform, Long Island City, NY, USA) according to conventional preparation guidelines (occlusal reduction of at least 1.5 mm, 4° to 8° taper, 1.5 mm axial reduction, 1.0 mm chamfer margin). An industrial-grade laboratory scanner (D2000; 3Shape, Copenhagen, Denmark) with an accuracy of 5 μm (ISO 12836) was used to scan the typodont three times. The scan files were imported into a digital inspection software, Geomagic Control X by 3D Systems (Rock Hill, SC, USA), and a master control file was created by taking the average of the three scans. The master scan was compared to the IOS scans to measure dimensional differences between the default standard-resolution scan and the high-resolution scan as obtained using the High res function.

### 2.2. Digital Impression Scan Preparation

**Figure 2:**
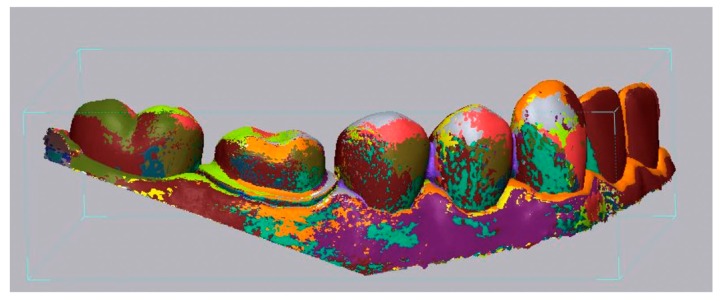
Superimposition of a master scan obtained from the 3Shape D2000 desktop scanner and intraoral scans from the 3Shape TRIOS 3 scanner using the Geomagic Control X software. The images were aligned using both the Initial Alignment and Best-Fit Alignment functions. The colors depict overlays of multiple scans.

**Figure 3:**
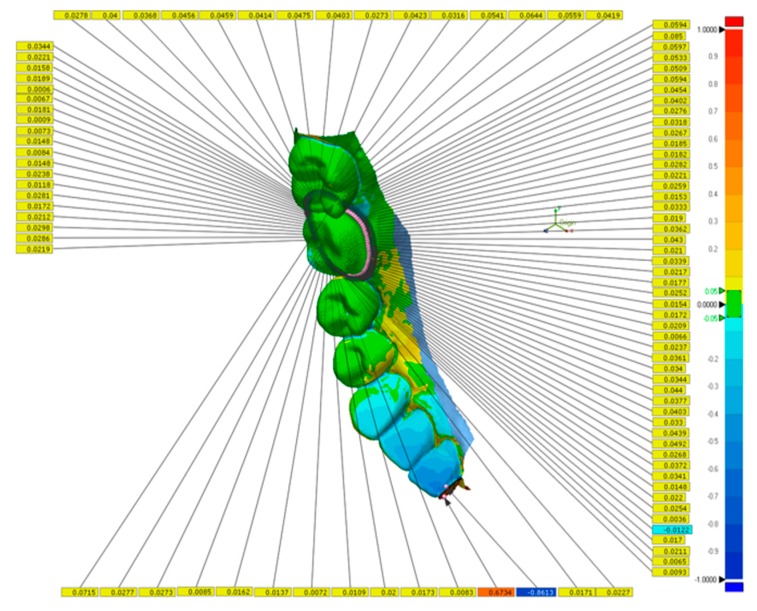
Deviation between master scan and intraoral scan measured at 100 evenly spaced points along the preparation finish line. The specific values of deviation along the finish line are given in yellow legends. The range of deviation across the entire half-arch scan is color graded from −1 mm (blue) to +1 mm (red).

A 3Shape TRIOS 3 intraoral scanner was used to produce digital impressions under three different resolution settings (n = 20 for each group): half lower-arch scan under standard resolution (SR group), half lower-arch scan under high resolution (HR group), and half lower-arch scan under standard resolution stitched with a high-resolution scan around the crown preparation margin (SHR group). Calibration of the scanner was performed prior to scanning according to the manufacturer’s guidelines. Scanning with the TRIOS 3 scanner was performed according to the procedures recommended by the user manual. The scans were performed by the same investigator on the same day to ensure consistency. The scan time for each individual scan and the number of images captured per scan were recorded. All 60 scans were imported into Geomagic Control X as stereolithography (STL) files, and initial alignment was applied to superimpose the IOS scans onto the master scan (Figure 2). Following initial alignment, all scans were cropped to the same size to eliminate artifacts and further optimize the uniformity of the scans. To measure the accuracy of scans around the preparation margin, the finish line was manually defined on the master scan using the “Curve” function of the software. A best-fit algorithm was applied to overlay each IOS scan to the master scan, and the 3D Compared tool was used to measure deviation at 100 evenly spaced locations on the previously defined finish line (Figure 3). In addition, the finish line was divided into four segments based on tooth surface: mesial, distal, buccal, and lingual. The buccal and lingual surfaces each accounted for 29 of the 100 points on the finish line, and the mesial and distal surfaces each accounted for 21 of the 100 points. The discrepancy for each tooth surface was calculated as the mean discrepancy of each corresponding segment.

### 2.3. Statistical Analysis

The number of samples to collect per group was determined by power analysis assuming a normal distribution and using data published for the closest set of 3Shape TRIOS 3 digital impressions by Ender et al. (2016) [12]. The total discrepancy, scan time, and number of images captured per scan were analyzed by one-way analysis of variance followed by multiple comparisons using Dunnett’s T3 test. Pearson correlations were calculated to correlate the total discrepancy with the scan time and number of images captured per scan. Discrepancies by tooth surface were analyzed by two-way repeated-measure analysis of variance with scan resolution and tooth surface as factors, followed by pairwise comparisons using the Bonferroni correction. All analyses were performed using the Statistical Package for Social Science (SPSS, Inc.) with the significance level set at α = 0.05.

## 3. Results

**Figure 4:**
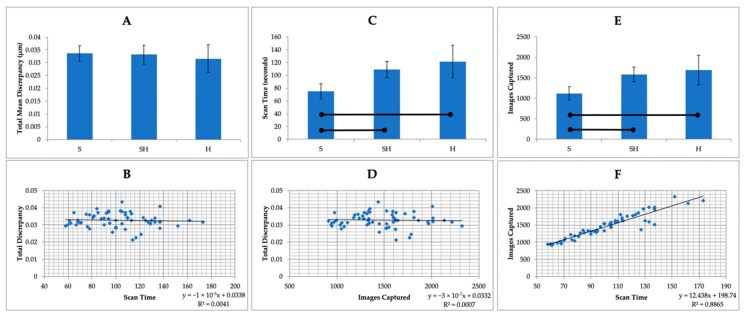
Comparison of total discrepancy, scan time, and number of images captured per scan between scan resolutions: standard resolution (SR), standard resolution with high resolution around the preparation margin (SHR), and high resolution (HR). Statistical analysis was performed using one-way analysis of variance followed by multiple comparisons using Dunnett’s T3 test. Pearson correlations were used to correlate total discrepancy with scan time and number of images captured. Horizontal bars show significant differences (*p* < 0.05). (**A**): total discrepancy by scan resolution: no statistically significant difference was observed in the overall comparison (*p* > 0.05). (**B**): no correlation was observed between total discrepancy and scan time. (**C**): a significant difference was observed between scan resolutions in regard to scan time, with the HR group having the longest mean scan time. (**D**): no correlation was observed between total discrepancy and numbers of images captured per scan. (**E**): a significant difference was observed between scan resolutions in regard to numbers of images captured per scan, with the HR group showing the highest number. (**F**): a positive correlation was seen between scan time and number of images captured per scan; the longer the scan time, the more images captured per scan.

On average, the scan times for the SHR group and the HR group were 34.2 s and 46.5 s longer compared to the SR group, respectively. The mean number of images captured per scan was 1124 for the SR group, 1584 for the SHR group, and 1692 for the HR group (Table 1). Scanning in high resolution in both the SHR and HR groups took significantly longer (*p* < 0.05) and required that more images be taken to complete the scan than for scanning in standard resolution alone (Figure 4C,E). No correlation was observed between total discrepancy and scan time or number of images captured (Figure 4B,D). Scan time and number of images captured showed a strong linear correlation (Figure 4F).

In total, 60 scans from three experimental groups were evaluated. Their accuracy was defined by measuring the discrepancy between the master scan and the IOS scans for the three scan groups. Discrepancy at the preparation finish line was calculated as the average distance between the master scan and the IOS scan at each of 100 points. For total discrepancy along the finish line, the HR group showed the lowest discrepancy value of 31.5 ± 5.5 μm, followed by the SHR group (33.2 ± 3.7 μm) and the SR group (33.6 ± 3.1 μm) (Table 2). These scanning discrepancies along the finish line were not significantly different among the three groups (*p* > 0.05; Figure 4A). 

**Figure 5:**
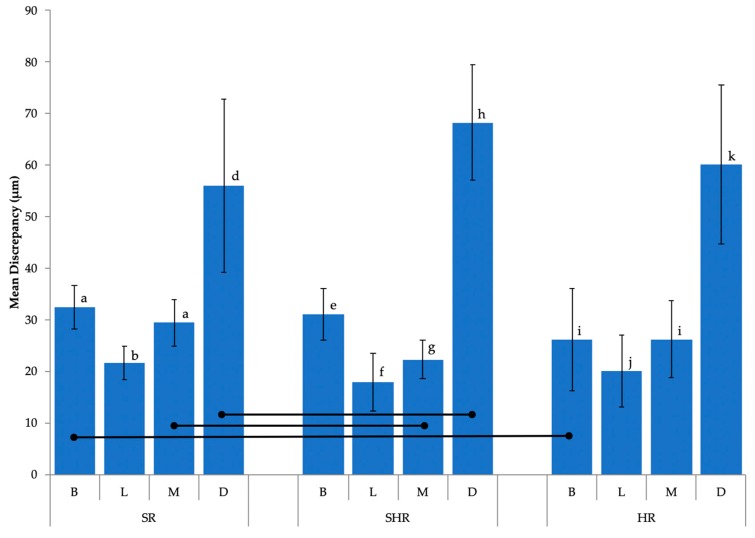
Mean discrepancy (μm) along the preparation finish line by tooth surface. Horizontal bars indicate significant differences (*p* < 0.05). Shared lowercase letters indicate no significant difference in discrepancy between tooth surfaces within groups. Distal surfaces showed significantly higher discrepancies when compared to other tooth surfaces across all three groups.

Additional statistical analysis by two-way repeated-measures ANOVA suggested that while scan resolution was not a significant factor (*p* > 0.05), significant differences were observed in discrepancy among tooth surfaces (*p* < 0.05) (Figure 5). Digital scanning of the distal surface was significantly less accurate when compared to that for the other three tooth surfaces in all three groups, with the discrepancy ranging from 56.1 ± 16.8 μm (SR group) to 68.2 ± 11.3 μm (SHR group) (Table 3).

## 4. Discussion

In this study, the accuracies of different scanning strategies using the 3Shape TRIOS 3 intraoral scanner were compared using an all-ceramic crown preparation of a mandibular first molar on a typodont. Differences in scanning accuracy at the preparation finish line between scan resolutions were examined to evaluate the necessity of including the additional step of scanning in High res mode to achieve optimal marginal integrity on the digital impression.

The null hypothesis that no significant difference would be observed among different intraoral scanning resolutions was not rejected by the results, suggesting that digitally trimming the preparation finish line and re-scanning in High res mode, which would increase the duration of a digital workflow, is not warranted. The additional time needed to digitally trim away the preparation margin and set up for High res mode in the software as well as the increased amount of computer data required (i.e., higher number of images taken) make this step in the scanning process undesirable. It is important to note that the scan time measured in this study was strictly based on the amount of time required to complete scanning as recorded by the software and did not take into account processing time in between scanning modes as well as the time needed to trim the margin, which would have further increased the total amount of time required for scanning when using the combination resolution technique.

Our results also revealed that the tooth surface plays a significant role in the accuracy of intraoral scans at the finish line. The lower accuracy observed for the distal surface suggests that interproximal regions where distances between adjacent teeth are small may be challenging for the current scanner, regardless of the scanning mode. A similar pattern was not observed for the mesial surface, however. The distance between the cavosurface margin of the prepared tooth and the adjacent tooth might impact the scanning accuracy. The absolute value of this distance on both mesial and distal surfaces should be measured in the future to evaluate the role that the distance between adjacent teeth surfaces plays in scanning accuracy. Another study reported that the crown preparation quality as measured by the tooth surface smoothness had a profound effect on the marginal fit of CAD/CAM-fabricated crowns, while the scanner type itself did not [13]. Furthermore, a previous in vivo study found that digital impression systems in general displayed increased distortion towards the distal end when full-arch impressions were taken [12]. This distortion pattern is consistent with the findings in this study.

Moreover, the occlusal plane slope and anatomy of the dental arch at the molar region could affect the distance of the scanner from the scanned margin, and therefore the focus of the obtained images. Additional studies should be conducted to examine the difference in scanning accuracy between surfaces of a single tooth at various positions in a dental arch.

The longevity and success of fixed dental prostheses depend heavily on marginal integrity between the preparation and the restoration. A systematic review conducted by Ahlholm et al. concluded that in their current state, digital impression techniques are clinically acceptable and comparable to conventional impression techniques in terms of accuracy for single crowns and short-span fixed partial dentures, but that their accuracy for complete dental arch treatment is lower [14]. The clinically acceptable value for marginal discrepancy of CAD/CAM-generated restorations has been described as between 50 and 100 μm [2,15]. This means that the accuracy of digital impression as the first step in any digital workflow should fall below that range in terms of marginal discrepancy. The results of this study showed that the accuracy of the 3Shape TRIOS 3 scanner, regardless of scanning resolution, falls within clinically accepted limits and is comparable to previously published data that showed a discrepancy that ranged from 6.9 ± 0.9 µm to 119 ± 48 μm [4,9,16,17,18,19,20]. The variation in observed discrepancy values could be due to differences in software and hardware versions, different materials scanned, operator discrepancies, differences in scanning strategies, or the span length of the scan.

Although numerous studies have compared the differences in scanning accuracy between various IOS, fewer studies have investigated the discrepancy in accuracy as a result of different scanning strategies. A common comparison between scanning protocols involves evaluating the difference in accuracy between strategies that differ in directional sequence. In an in vitro study, Müller et al. found that changing the directional sequence of scanning did not impact the accuracy of a full-arch digital impression using the 3Shape TRIOS Pod scanner [21]. Furthermore, Medina-Sotomayor et al. explored how differences in scanning direction affected the accuracy of digital impressions using four different IOS and found no significant difference among scanning systems [16]. It is important to note, however, that scanning strategies based on the sequence in which a tooth surface is scanned could be utilized by other intraoral scanning systems, thus allowing for a comparison between scanners to be made. Conversely, it is to the best of the authors’ knowledge that the high-resolution scanning tool investigated in this study is a unique feature of the 3Shape TRIOS scanning interface, and a scanning protocol combining different optical resolutions has not been recommended by other manufacturers.

In addition, 3Shape has recently released a new scanner model, the TRIOS 4 scanner, with a Zoom function that appears to be similar in functionality to the TRIOS 3 High res/Zoom setting. As intraoral scanners and associated software are developed, it is possible that the accuracy and precision of impressions can improve substantially. Further studies are needed to fairly evaluate the TRIOS 4 scanner and its optical resolution. The authors plan to utilize the methodology developed in the current study to further examine the TRIOS 4 scanner, as well as scanning parameters recommended by other major intraoral scanners comprehensively in the near future.

Previous studies have been performed to examine the difference in accuracy according to multiple scan strategies within a single scanning system. Motel et al. compared the difference in accuracy between two strategies for implant impression using the TRIOS 3 scanner in an in vitro study [22]. The first strategy involved a one-step approach by scanning both the scan bodies and surrounding structures together, whereas the second strategy combined an initial scan without the scan bodies and a final scan with the scan bodies in place. These authors concluded that the one-step scanning strategy achieved significantly higher accuracy compared to the two-step approach. This finding is consistent with our recommendation of a one-step scanning approach for optimal scanning efficiency.

The 3Shape TRIOS 3 intraoral scanner was selected for this study due to its popularity and proven superior accuracy. Accordingly, a limitation of our study is that it was designed specifically for the TRIOS 3 scanner and other IOS were not included for comparison. Therefore, our conclusion of no significant difference in digital impression accuracy between default resolution and high resolution using TRIOS 3 should not be considered applicable for other IOS without further evaluation. While the scope of this study limits its direct application to users of TRIOS 3, the results of this study highlight the importance of evaluating different parameters within a single scanning system when making digital impressions for the fabrication of dental prostheses.

Given that this was an in vitro study, factors that could influence the scanning accuracy when digital impressions are taken in vivo, such as the presence of saliva and blood, soft tissue movement, and the limited space that the oral cavity allows for maneuvering of the camera, were not considered. The notion that the High res function could yield superior accuracy when such in vivo artifacts are present is plausible. Further study goals include the investigation of the impact of intraoral variables and margin design on scanning accuracy under different optical resolutions in clinical settings.

## 5. Conclusions

Within the limitations of this in vitro study, the following conclusions were made:

1. Significant differences in terms of scan time and number of images captured per scan were observed among the three groups with different scanning resolution settings.
2. No significant difference was observed between default resolution and high resolution in terms of accuracy on the crown preparation cavosurface finish line using the 3Shape TRIOS 3 intraoral scanner.
3. Scanning accuracy was significantly affected by tooth surface, with the distal surface demonstrating the lowest accuracy.

## Figures and Tables

**Table 1:** Mean scan time (seconds) and average number of images captured per scan.

Group	Scan Time (s)	Images Captured
**SR**	75.05 ± 11.7	1124 ± 161
**SHR**	109.25 ± 12.6	1584 ± 179
**HR**	121.5 ± 25.5	1692 ± 358

**Table 2:** Total mean discrepancy (μm) by scan resolution.

Group	Total Discrepancy (μm)
**SR**	31.5 ± 5.5
**SHR**	33.2 ± 3.7
**HR**	33.6 ± 3.1

**Table 3:** Mean discrepancy by tooth surface (μm).

Scan Resolution	Discrepancy by Surface (μm)			
	Buccal	Lingual	Mesial	Distal
**SR**	32.5 ± 4.2	21.6 ± 3.2	29.4 ± 4.5	56.1 ± 16.8
**SHR**	31.0 ± 4.9	17.9 ± 5.6	22.3 ± 3.8	68.2 ± 11.3
**HR**	26.1 ± 9.9	20.1 ± 7.0	26.2 ± 7.4	60.1 ± 15.5
